# Plasmacytoid Dendritic Cells as a New Therapeutic Target for Autoimmune Pancreatitis and IgG4-Related Disease

**DOI:** 10.3389/fimmu.2021.713779

**Published:** 2021-07-23

**Authors:** Kosuke Minaga, Tomohiro Watanabe, Akane Hara, Tomoe Yoshikawa, Ken Kamata, Masatoshi Kudo

**Affiliations:** Department of Gastroenterology and Hepatology, Kindai University Faculty of Medicine, Osaka-Sayama, Japan

**Keywords:** autoimmune pancreatitis, IgG4-related disease, plasmacytoid dendritic cells, interferon-I, interleukin-33

## Abstract

Although plasmacytoid dendritic cells (pDCs) able to produce large amounts of type 1 interferons (IFN-I) play beneficial roles in host defense against viral infections, excessive activation of pDCs, followed by robust production of IFN-I, causes autoimmune disorders including systemic lupus erythematosus (SLE) and psoriasis. Autoimmune pancreatitis (AIP), which is recognized as a pancreatic manifestation of systemic immunoglobulin G4-related disease (IgG4-RD), is a chronic fibroinflammatory disorder driven by autoimmunity. IgG4-RD is a multi-organ autoimmune disorder characterized by elevated serum concentrations of IgG4 antibody and infiltration of IgG4-expressing plasmacytes in the affected organs. Although the immunopathogenesis of IgG4-RD and AIP has been poorly elucidated, recently, we found that activation of pDCs mediates the development of murine experimental AIP and human AIP/IgG4-RD *via* the production of IFN-I and interleukin-33 (IL-33). Depletion of pDCs or neutralization of signaling pathways mediated by IFN-I and IL-33 efficiently inhibited the development of experimental AIP. Furthermore, enhanced expression of IFN-I and IL-33 was observed in the pancreas and serum of human AIP/IgG4-RD. Thus, AIP and IgG4-RD share their immunopathogenesis with SLE and psoriasis because in all these conditions, IFN-I production by pDCs contributes to the pathogenesis. Because the enhanced production of IFN-I and IL-33 by pDCs promotes chronic inflammation and fibrosis characteristic for AIP and IgG4-RD, neutralization of IFN-I and IL-33 could be a new therapeutic option for these disorders. In this Mini Review, we discuss the pathogenic roles played by the pDC-IFN-I-IL-33 axis and the development of a new treatment targeting this axis in AIP and IgG4-RD.

## Introduction

Plasmacytoid dendritic cells (pDCs) were initially identified over two decades ago as a unique subset of dendritic cells that can produce abundant quantities of type 1 interferons (IFN-I) (1). Although pDCs constitute a very small percentage of human and murine immune cells (2), this cell type is a major cellular source of IFN-I and plays critical roles in host defense against microbial infection. This idea is supported by the findings that mice lacking pDCs or those treated with a pDC-depleting antibody (Ab) exhibit defective IFN-I responses (3). Activation of pDCs, followed by enhanced IFN-I production, is essential for the initiation of innate immune responses against viral infections (3, 4). Recent reports indicate that during infection with severe acute respiratory syndrome coronavirus 2, which has recently caused a pandemic worldwide, host immune defenses involve pDC activation (5). However, excessive production of IFN-I by pDCs also underlies the immunopathogenesis of a broad range of autoimmune disorders (6). Typical autoimmune diseases driven by the activation of pDCs include systemic lupus erythematosus (SLE) (7, 8), psoriasis (9), and type 1 diabetes (T1D) (10, 11). A recent clinical trial, in which patients with active SLE were successfully treated with biologics targeting IFN-I, verified the pathogenic roles of IFN-I produced by pDCs (12–14).

Type 1 autoimmune pancreatitis (AIP), which is recognized as a pancreatic manifestation of systemic immunoglobulin G4-related disease (IgG4-RD), is a chronic fibroinflammatory disorder of the pancreas (15–18). In this article, type 1 AIP is hereafter referred to as ‘AIP’. IgG4-RD and AIP are newly established multi-organ autoimmune disorders characterized by elevated serum concentrations of IgG4 Ab and infiltration of IgG4-expressing plasmacytes into the affected organs. Although some of the molecular mechanisms accounting for enhanced IgG4 Ab responses are being elucidated, their immunopathogenesis remains poorly understood. Recently, we found that activation of pDCs mediates the development of murine AIP and human AIP/IgG4-RD *via* the production of IFN-I and interleukin-33(IL-33) (17, 19–23). Depletion of pDCs or neutralization of signaling pathways mediated by IFN-I and IL-33 efficiently inhibited the development of experimental AIP. Furthermore, enhanced expression of IFN-I and IL-33 was observed in the pancreas and serum of patients with AIP and IgG4-RD. Moreover, AIP and IgG4-RD share the mechanism of their immunopathogenesis with other autoimmune diseases, including SLE and psoriasis, in that the autoimmunity is caused by IFN-I production by pDCs. However, the IL-33-mediated signaling pathway is activated only in AIP and IgG4-RD, but not in SLE or psoriasis. Given that enhanced production of IFN-I and IL-33 by pDCs promotes chronic inflammation and fibrosis, which are characteristic features of AIP and IgG4-RD, neutralization of IFN-I and IL-33 could be a new therapeutic option for these disorders. In this Mini Review, we discuss pathogenic roles played by the pDC-IFN-I-IL-33 axis and propose novel treatments targeting this axis in AIP and IgG4-RD.

## IFN-I Signaling Pathways in pDCs

Innate immune responses initiated by Toll-like receptors (TLRs) are critical for host defense against pathogens (24). pDCs preferentially express endosomal TLR7 and TLR9, which detect single-stranded RNA and double-stranded DNA derived from bacteria and viruses (24). Innate immune responses mediated by TLR7 and TLR9 depend upon the activation of myeloid differentiation primary response protein 88 (MyD88). The interaction between TLR7/9 and MyD88 is followed by the activation of interleukin-1-receptor-associated kinase 4 (IRAK4) (24, 25). The kinase activity of IRAK4 mediates the formation of complexes consisting of IRAK1, tumor-necrosis factor receptor-associated factor 3 (TRAF3), TRAF6, inhibitor of NF-κB kinase α (IKKα), and interferon regulatory factor 7 (IRF7) (4, 24, 25). Formation of this complex leads to the nuclear translocation of IRF7, a critical transcription factor for the initial production of IFN-I in pDCs (24–26). IRF7, which is polyubiquitinated by TRAF6 after interaction with MyD88 (27), is the master regulator of IFN-I production. This idea is fully supported by studies showing that pDCs isolated from mice deficient in IRF7 or MyD88 exhibit defective IFN-I production upon stimulation with TLR9 ligands (28).

As mentioned above, sensing of single-stranded RNA and double-stranded DNA by endosomal TLR7 and TLR9 induces the activation of the MyD88-IRAK4-IRAK1-TRAF6-TRAF3-IKKα-IRF7 pathway, thereby leading to the initial production of IFN-I by pDCs (24–26). Although the initial production of IFN-I is low, IFN-I-mediated signaling pathways are augmented by the presence of a positive feedback loop (26, 29). IFN-I activates the cell surface IFN-I receptor followed by nuclear translocation of IFN-stimulated gene factor 3, which is composed of signal transduction and activator of transcription 1 (STAT1), STAT2, and IRF9 (26, 29), and promotes the transcription of IRF7 by binding to its putative promoter regions. The newly synthesized IRF7, in turn, leads to the amplification of IFN-I transcription. This positive feedback loop of IFN-I response is useful for the eradication of viruses and bacteria; however, it may also augment IFN-I responses associated with autoimmunity.

## Pathogenic Roles of pDCs in Autoimmune Diseases

Although pDCs play beneficial roles in host defense against viral infections, excessive activation of pDCs, followed by robust production of IFN-I, causes autoimmune diseases. SLE is the most well-studied autoimmune disease the pathogenesis of which is significantly affected by the pDC-IFN-I axis (30). This notion is supported by the finding that elevated serum concentrations of IFN-I observed in patients with SLE correlate with both disease activity and severity (31). SLE is a chronic multi-organ disorder characterized by the production of Abs to self-nucleic acids and by the deposition of immune complexes (32, 33). In SLE, immune complexes composed of self-nucleic acids and antinuclear Abs are efficiently taken up by cell surface Fc receptors and then delivered into the endosomal components of pDCs (3, 34, 35). Sensing of self-nucleic acids by endosomal TLR7 and TLR9 results in IFN-I production by pDCs. Thus, activation of TLR7 and/or TLR9 by self-nucleic acids is an indispensable step for pDC-mediated IFN-I responses in SLE.

**Figure 1:**
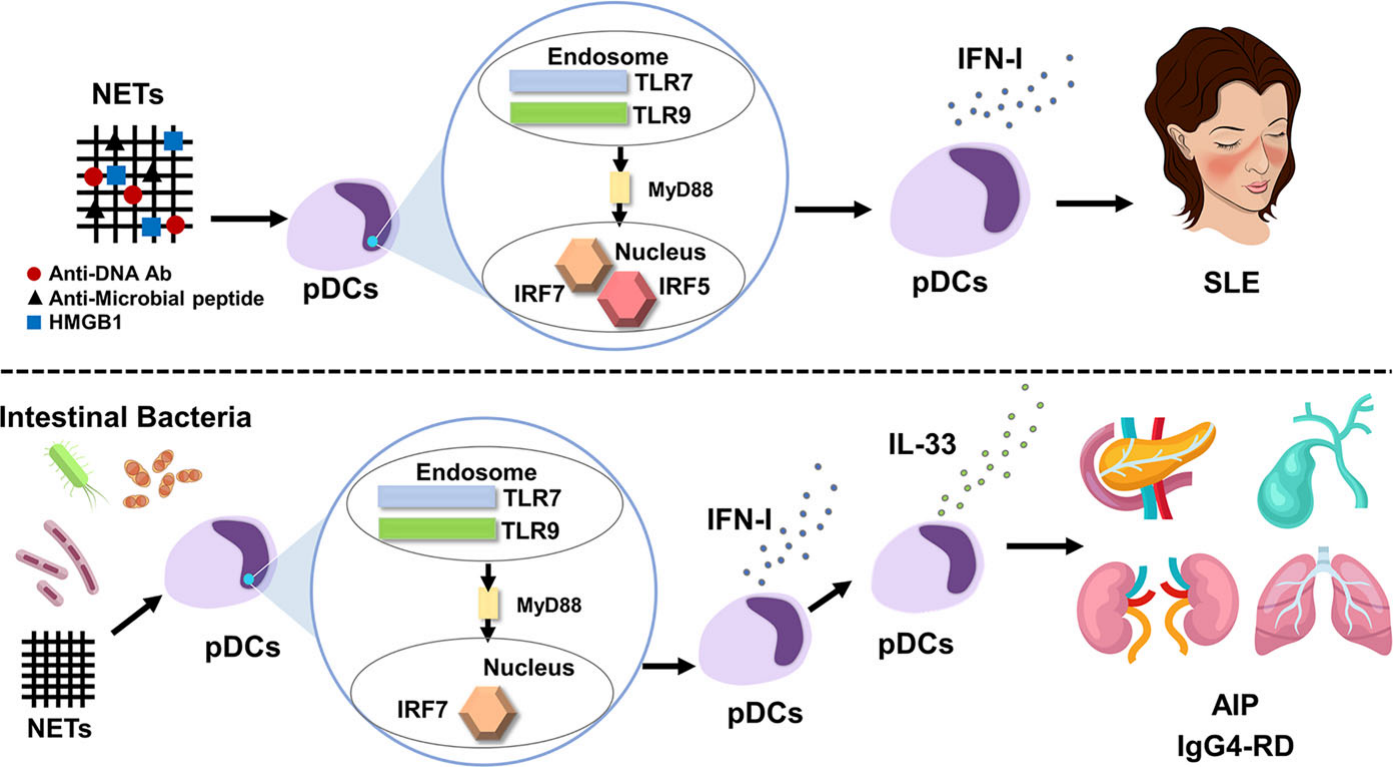
IFN-I produced by plasmacytoid dendritic cells mediates systemic lupus erythematosus and autoimmune pancreatitis/IgG4-related disease. Neutrophil extracellular traps (NETs) activate plasmacytoid dendritic cells (pDCs) to produce large amounts of IFN-I. pDC-mediated IFN-I responses underlie the immunopathogenesis of systemic lupus erythematosus (SLE). Myeloid differentiation primary response protein 88 (MyD88)-dependent activation of Toll-like receptor 7 (TLR7) and TLR9 induces IFN-I production through nuclear translocation of interferon regulatory factor 5 (IRF5) and IRF7 (top panel). Intestinal dysbiosis and NETs activate pDCs to produce large amounts of IFN-I through nuclear translocation of IRF7, which, in turn, results in the enhanced production of IL-33 by pDCs. The pDC-IFN-I-IL-33 axis underlies the immunopathogenesis of autoimmune pancreatitis (AIP) and IgG4-related disease (IgG4-RD) (bottom panel).

Regarding the trigger for IFN-I production by pDCs, recent studies have highlighted the importance of neutrophil extracellular traps (NETs), web-like structures released by activated neutrophils (36–39). NETs, composed of chromatin DNA, oxidized mitochondrial DNA, antimicrobial peptides, and the high-mobility group box 1 (HMGB1) protein, can function as a trigger for excessive IFN-I secretion through the activation of TLR7 and/or TLR9 in pDCs of patients with SLE (36–39). In fact, increased formation of NETs and elevated concentrations of NET components, such as antimicrobial peptides and HMGB1, were observed in the serum of patients with SLE as compared with those in healthy controls (37). Thus, enhanced IFN-I production caused by NET-mediated TLRs activation underlies the immunopathogenesis of human SLE (**Figure 1**). As for transcription factors for IFN-I production involved in the immunopathogenesis of SLE, recent studies highlight the importance of IRF5 in parallel to IRF7 (40, 41). *IRF5* gene polymorphisms associated with SLE cause enhanced expression of IRF5 and hyperactivation of IRF5 underlies the immunopathogenesis of SLE through induction of IFN-I production (40, 41).

In line with human studies, experimental murine models of lupus provided evidence supporting the immunopathogenicity of pDCs. NZB and BXSB mice spontaneously develop murine lupus (30). Neutralization of the IFN-I receptor in BXSB mice and genetic deletion of the IFN-I receptor in NZB mice protected these mouse strains from the development of lupus, suggesting that spontaneous development of murine lupus required intact IFN-I signaling pathways (42, 43). Recent studies have successfully shown that pDC-mediated IFN-I responses cause experimental murine lupus. To investigate the specific contribution of pDCs in murine lupus, transgenic mice were created that expressed the diphtheria toxin (DT) receptor under the control of the highly specific human pDC *CLEC4C*/*BDCA2* promoter. Administration of DT before disease onset inhibited the development of lupus by selective systemic ablation of pDCs, which was accompanied by impaired expression of genes stimulated by IFN-I (44). Interestingly, these beneficial effects of transient pDC depletion were sustained even after pDC recovery, indicating crucial roles of pDC-mediated IFN-I responses in disease initiation (44). This idea was supported by another study in mice with impairment of pDC function caused by monoallelic deletion of the pDC-specific transcription factor E2-2. Sisirak et al. reported that impairment of pDC function resulted in the amelioration of murine lupus caused by the overexpression of TLR7 (45). These animal studies confirmed the concept that TLR-mediated IFN-I responses by pDCs play crucial roles in the development of both human SLE and murine lupus.

The pDC-IFN-I axis has been implicated in psoriasis development. Psoriasis is the most common autoimmune disease of the skin and is characterized by the infiltration of immune cells and hyperproliferation of keratinocytes (46). The accumulation of pDCs expressing IFN-I and IRF7 was much greater in the skin of patients with psoriasis than in those of healthy controls (9). Sensing of self-DNA coupled with antimicrobial peptides by TLR9 induces IFN-I production by pDCs residing in the skin of patients with psoriasis (47). Glitzner et al. directly addressed the role of pDCs in the development of experimental psoriasis by crossing *Jun* ^f/f^ *JunB* ^f/f^ *K5cre-ER^T^* mice with *BDCA2-DTR* mice to deplete pDCs (48). They found that the depletion of pDCs by DT injection resulted in the amelioration of experimental psoriasis, which was accompanied by downregulation of IL-23 expression (48). Thus, they provide evidence that activation of pDCs mediates psoriatic lesions by enhancing signaling pathways mediated not only by IFN-I but also by IL-23. The latter cytokine plays a crucial role in the development of psoriasis, as evidenced by the fact that biologics targeting IL-23 are very effective in patients with psoriasis (49). In addition to psoriasis, IFN-I production by pDCs plays pathogenic roles in the development of T1D (10, 11). In these studies, activation of pDCs by self DNA, DNA-specific IgG, and antimicrobial peptide induces IFN-I production through TLR9 in the pancreatic islets (10, 11).

## pDCs and Autoimmune Pancreatitis/IgG4-Related Disease

Although AIP and IgG4-RD are characterized by enhanced adaptive immune responses that include the IgG4 Ab response, recent studies have shed light on the presence of innate immune responses as well. Repeated injection of polyinosinic:polycytidylic acid (poly (I:C)) into MRL/MpJ mice leads to the development of AIP, autoimmune sialadenitis, cholangitis, and glomerulonephritis, all of which are organ-specific manifestations of AIP and IgG4-RD (50). Extensive flow cytometry analyses performed using pancreatic immune cells found massive accumulation of pDCs in the pancreas of MRL/MpJ mice displaying AIP. Consistent with the pancreatic accumulation of pDCs, IFN-I expression was markedly enhanced in the pancreas of MRL/MpJ mice (19). The development of experimental AIP was dependent upon the activation of pDC-mediated IFN-I signaling pathways because the administration of pDC-depleting or IFN-I neutralizing Abs efficiently prevented the development of experimental AIP (19). Administration of the IRF7-specific siRNA almost completely prevented the development of experimental AIP through the downregulation of IFN-I expression, suggesting that experimental AIP required the nuclear translocation of IRF7 (21).

A specific type of fibrosis called storiform fibrosis is one of the characteristic pathological findings in AIP and IgG4-RD (15–18). IL-33 produced by pancreatic acinar cells induces chronic fibroinflammatory responses in experimental chronic alcoholic pancreatitis (51, 52). As in the case of chronic pancreatitis, the pancreatic expression of IL-33 is much greater in AIP mice than in normal mice (20). Interestingly, cell purification and cell depletion studies have revealed that pDCs are a cellular source of IL-33 (20). IL-33 that is produced by pDCs in an IFN-I dependent manner is necessary for the development of chronic fibroinflammatory responses in the pancreas, as is shown by the neutralization of IL-33-mediated signaling pathways and attenuation of experimental AIP by using an anti-ST2 Ab (20). Although, pro-IL-33 is proteolytically activated into a bioactive form by caspase-1, 3, 7, it remains unknown whether caspase-mediated processing is operating in IL-33 production by pDCs in AIP and IgG4-RD (53).

The clinical relevance of these data in experimental AIP has been verified in human samples from patients with AIP and IgG4-RD. pDCs expressing IRF7, IFN-I, and IL-33 accumulated in the pancreas of patients with AIP and IgG4-RD (19–21). Moreover, peripheral blood pDCs isolated from patients with AIP and IgG4-RD promoted IgG4 Ab production by naïve B cells present in the peripheral blood of healthy controls in an IFN-I-dependent and T cell-independent manner (19). Thus, these studies support the idea that pDC-mediated production of IFN-I and IL-33 underlies the immunopathogenesis of AIP and IgG4-RD. We recently identified serum concentrations of IFN-I and IL-33 as novel biomarkers for AIP and IgG4-RD (54). Serum concentrations of these two cytokines were much higher in patients with AIP and IgG4-RD than in those with chronic pancreatitis or healthy controls. In addition, the induction of remission by prednisolone (PSL) was associated with a marked reduction in serum concentrations of IFN-I and IL-33 in patients with AIP and IgG4-RD. Thus, IFN-I and IL-33 produced by pDCs are also useful as biomarkers in the clinical identification of patients with AIP and IgG4-RD.

NETs and intestinal dysbiosis have been implicated as possible triggers of pDC activation in AIP and IgG4-RD. NETs formation was confirmed in the pancreas of MRL/MpJ mice displaying AIP and in patients with AIP and IgG4-RD (19). In addition to NETs, intestinal dysbiosis also mediates pDC activation in experimental AIP. Bowel sterilization by antibiotics completely prevented the development of experimental AIP, which was accompanied by reduced activation of pDCs expressing IFN-I and IL-33 (22). Repeated injections of 10 μg and 100 μg poly (I:C) into MRL/MpJ mice induced mild and severe types of AIP, respectively (22). We took advantage of the relationship between poly (I:C) doses and AIP severity and then performed co-housing and fecal microbiota studies. As expected, mice treated with 10 μg of poly (I:C) developed a mild degree of AIP. Interestingly, mice treated with 10 μg of poly (I:C) developed severe AIP equivalent to that induced by injection of 100 μg of poly (I:C) upon co-housing with mice treated with 100 μg of poly (I:C) or when they were exposed to fecal microbiota from donor mice treated with 100 μg of poly (I:C) (22). Such development of severe AIP was associated with enhanced pancreatic accumulation of pDCs producing IFN-I and IL-33. Thus, these studies provide evidence that intestinal dysbiosis mediates the development of experimental AIP through the activation of pDCs (**Figure 1**). In line with the results of experimental AIP, alterations in fecal microbiota composition were observed in patients with AIP (23). Disappearance of *Klebsiella pneumoniae* was observed in the stool of two of three patients with AIP after successful induction of remission by PSL. Mice treated with 10 μg of poly (I:C) in combination with oral administration of heat-killed *K. pneumoniae* developed more severe AIP as compared with the condition of mice treated with poly (I:C) or *K. pneumoniae* alone. Severe AIP induced by co-administration of poly (I:C) and *K. pneumoniae* was associated with increased accumulation of pDCs producing IFN-I and IL-33. Taken together, these findings suggest that intestinal dysbiosis mediates AIP through the activation of pDCs producing IFN-I and IL-33. However, it should be noted that cellular sources of IL-33 are not limited to pDCs (55–57). In particular, M2 macrophages present in the salivary glands have been identified as potent producers of IL-33. As for possible triggers for pDC activation in human AIP and IgG4-RD, NETs formation was observed in the pancreas of patients with IgG4-associated AIP (19). Moreover, intestinal dysbiosis was associated with the induction of remission in patients with AIP (23). Therefore, NETs and intestinal dysbiosis may function as possible triggers for pDC activation.

It is well established pDCs preferentially activate regulatory T cells (Tregs) (58, 59). If pDCs are abundant in the affected organs of AIP and IgG4-RD, then activation of Tregs is induced in the lesions of AIP and IgG4-RD. In fact, chronic inflammatory lesions of AIP and IgG4-RD are characterized by accumulation of Tregs (17).

## pDCs as a Therapeutic Target in Autoimmune Diseases

**Figure 2:**
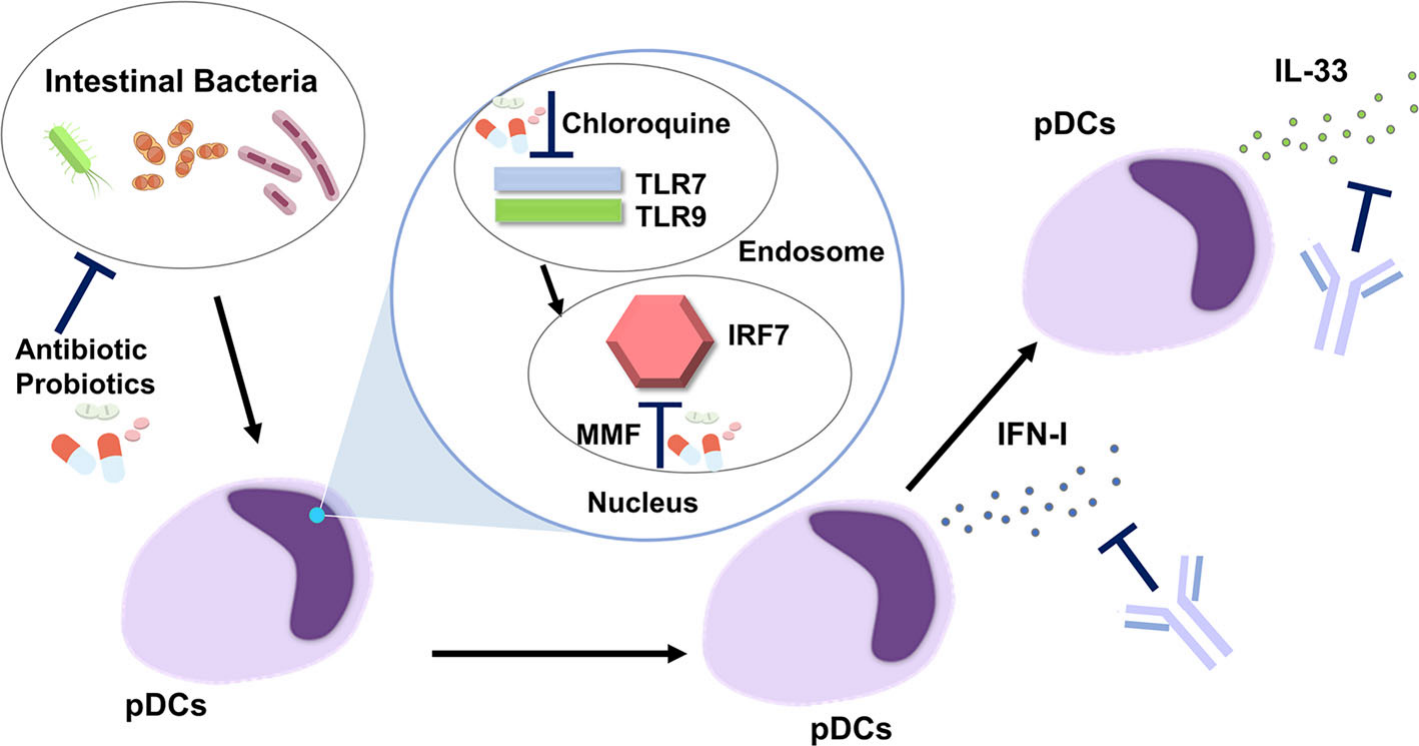
Development of new treatments targeting the plasmacytoid dendritic cell-IFN-I-IL-33 axis in autoimmune pancreatitis and IgG4-related disease. Intestinal dysbiosis activates endosomal Toll-like receptor 7 (TLR7) and TLR9 followed by nuclear translocation of IFN regulatory factor 7 (IRF7). pDCs produce IFN-I through the nuclear translocation of IRF7. IL-33 is produced by pDCs in an IFN-I-dependent manner. Antibiotics and probiotics may be useful for correction of intestinal dysbiosis. Chloroquine inhibits the activation of endosomal TLR7 and TLR9. Mycophenolate mofetil (MMF) suppresses nuclear translocation of IRF7. IFN-I-mediated signaling pathways are efficiently inhibited by Abs against IFN-I or IFN-I receptor. An anti-ST2 Ab, neutralizing the IL-33 receptor, blocks IL-33-mediated signaling pathways.

Clinical success targeting the pDC-IFN-I axis in SLE led us to hypothesize that patients with AIP and IgG4-RD can be successfully treated by blocking this pathway (**Figure 2**). As in the case of SLE, neutralization of IFN-I by anifrolumab or sifalimumab may be effective in patients with AIP and IgG4-RD (12–14). In contrast to the case with SLE, IL-33 produced by pDCs can be another treatment target for AIP and IgG4-RD. Serum concentrations of IFN-I and IL-33 have been identified as novel biomarkers useful for the diagnosis and evaluation of disease activity in patients with AIP and IgG4-RD, whereas serum concentrations of the latter cytokine were comparable in patients with SLE and healthy controls (54, 60, 61). Therefore, an anti-ST2 Ab or etokimab (62) targeting IL-33-mediated signaling pathways might be a unique therapeutic option for patients with AIP and IgG4-RD. This notion is supported by the finding that the blockade of IL-33-mediated signaling pathways by anti-ST2 Ab prevented not only fibrogenesis but also inflammation in experimental AIP (20). Thus, biologics targeting IFN-I and IL-33 may be promising therapeutics in patients with AIP and IgG4-RD, as evidenced by the results of animal studies, in which the neutralization of IFN-I or IL-33-mediated signaling pathways by Abs efficiently prevented the development of experimental AIP.

In addition to the IFN-I-IL-33 axis, correction of intestinal dysbiosis by antibiotics or probiotics might be useful for the suppression of pDC activation. This idea is supported by the fact that bowel sterilization by a broad range of antibiotics almost completely prevented the development of experimental AIP (22). The intracellular signaling pathway involves the activation of endosomal TLR7 and TLR9, followed by nuclear translocation of IRF7, to initiate the transcription of IFN-I. In the case of SLE, chloroquine, a potent inhibitor of endosomal activation of TLR7 and TLR9, has been shown to offer a survival advantage (33). Moreover, mycophenolate mofetil, another inhibitor of IRF7 (63), is a first-line therapy in the management of lupus nephritis and cutaneous disease (33). Thus, chloroquine and mycophenolate mofetil may be effective for patients with AIP and IgG4-RD as they would inhibit signaling pathways mediated by the activation of TLR7, TLR9, and IRF7.

In most cases of AIP and IgG4-RD, PSL is very effective for the induction of remission (15–18). It should be noted, however, that a significant fraction of patients with AIP and IgG4-RD experience repeated episodes of relapse, even upon standard treatment with PSL. Moreover, treatment with PSL can cause severe side effects such as diabetes mellitus, opportunistic infections, and osteoporosis. The new treatment targeting the pDC-IFN-I-IL-33 axis may be useful for such patients. Rituximab therapy is useful for patients with IgG4-RD (64). However, it is poorly understood whether induction of remission by rituximab is accompanied by reduction in IFN-I-IL-33 responses.

## Conclusion

AIP and IgG4-RD are characterized by the activation of pDCs producing IFN-I and IL-33. Serum concentrations of IFN-I and IL-33 have been identified as novel biomarkers for AIP and IgG4-RD. Targeting the IFN-I-IL-33 axis in pDCs might constitute a successful approach to treat patients with AIP and IgG4-RD, especially those who suffer from repeated episodes of relapse even with PSL treatment or side effects associated with PSL.

## Author Contributions

KM and TW drafted the manuscript and prepared the figures. AH, TY, and KK reviewed the manuscript for its intellectual content. KM, TW, and MK were responsible for revising the manuscript. All authors contributed to the article and approved the submitted version.

## Funding

This work was supported by Grants-in-Aid for Scientific Research (19K08455, 19K17506, 20K16975, 21K15987) from the Japan Society for the Promotion of Science, Takeda Science Foundation, Yakult Bio-Science Foundation, SENSHIN Medical Research Foundation, and Japan Agency for Medical Research and Development (AMED) for Research on Intractable Diseases.

## Conflict of Interest

The authors declare that the research was conducted in the absence of any commercial or financial relationships that could be construed as a potential conflict of interest.

## Publisher’s Note

All claims expressed in this article are solely those of the authors and do not necessarily represent those of their affiliated organizations, or those of the publisher, the editors and the reviewers. Any product that may be evaluated in this article, or claim that may be made by its manufacturer, is not guaranteed or endorsed by the publisher.

## Abbreviations

pDCs, Plasmacytoid dendritic cells; IFN-I, Interferon-I; SLE, Systemic lupus erythematosus; AIP, Autoimmune pancreatitis; IgG4-RD, Immunoglobulin G4-related disease; IL-33, Interleukin-33; TLR, Toll-like receptor; MyD88, Myeloid differentiation primary response protein 88; IRAK4, Interleukin-1-receptor-associated kinase 4; TRAF, Tumor-necrosis factor receptor-associated factor; IKKα, Inhibitor of NF-κB kinase α; IRF, Interferon regulatory factor; STAT, Signal transduction and activator of transcription; NETs, Neutrophil extracellular traps; HMGB1, High-mobility group box 1; DT, Diphtheria toxin; poly (I:C), polyinosinic:polycytidylic acid; PSL, Prednisolone; T1D, Type 1 diabetes; Treg, Regulatory T cell.
